# Preoperative Radiographic Thoracic Kyphosis Relates to Scapular Internal Rotation but Not Anterior Tilt in Candidates for Reverse Shoulder Arthroplasty: A Retrospective Radiographic Analysis from the FP-UCBM Shoulder Study Group

**DOI:** 10.3390/jcm14228183

**Published:** 2025-11-18

**Authors:** Edoardo Franceschetti, Pietro Gregori, Chiara Capperucci, Mauro La Bruna, Giancarlo Giurazza, Andrea Tanzilli, Michele Paciotti, Cirino Amato, Umile Giuseppe Longo, Rocco Papalia

**Affiliations:** 1 Fondazione Policlinico Universitario Campus Bio-Medico, Via Alvaro del Portillo, 200, 00128 Roma, Italy; e.franceschetti@policlinicocampus.it (E.F.); chiara.capperucci@unicampus.it (C.C.); maurolabruna94@gmail.com (M.L.B.); g.giurazza@unicampus.it (G.G.); andrea.tanzilli@unicampusus.it (A.T.); m.paciotti@policlinicocampus.it (M.P.); r.amato@policlinicocampus.it (C.A.); g.longo@policlinicocampus.it (U.G.L.); r.papalia@policlinicocampus.it (R.P.); 2 Research Unit of Orthopaedic and Trauma Surgery, Department of Medicine and Surgery, Università Campus Bio-Medico di Roma, Via Alvaro del Portillo, 21, 00128 Roma, Italy

**Keywords:** thoracic kyphosis, shoulder function, scapular internal rotation, anterior scapular tilt, reverse shoulder arthroplasty

## Abstract

**Background/Objectives**: In the elderly population, thoracic kyphosis often progresses with age, leading to secondary postural adaptations including scapular protraction, internal rotation, and anterior tilt. These alterations can potentially compromise shoulder biomechanics, particularly in patients undergoing reverse shoulder arthroplasty (RSA). The purpose of this study was to evaluate the relationship between thoracic sagittal alignment, quantified by the Cobb angle, and scapular internal rotation (SIR) assessed on CT scans in patients scheduled for RSA. **Methods**: A retrospective study was conducted on 164 patients who underwent RSA between 2016 and 2024 at a single tertiary referral center. Sagittal thoracic kyphosis was assessed using the Cobb angle measured on preoperative chest radiographs. SIR and anterior scapular tilt were evaluated using preoperative CT scans. Patients were divided into three groups according to the Cobb angle: Group A (≤36°), Group B (>36–46°), and Group C (≥47°). Statistical analysis was performed using the Spearman correlation coefficient and Kruskal–Wallis test, with a significance threshold set at *p* < 0.05. **Results**: Analysis demonstrated a weak but statistically significant positive correlation between age and SIR, as well as between thoracic kyphosis (Cobb angle) and SIR. Patients in Group C (Cobb angle ≥ 47°) showed higher mean SIR values (43.7°) compared to Group A (40.3°), with statistical significance achieved (*p* = 0.047). These findings suggest that greater thoracic kyphosis is associated with increased scapular internal rotation. No significant correlation was identified between anterior scapular tilt and thoracic kyphosis. **Conclusions**: This study reveals a correlation between increased thoracic kyphosis and greater scapular internal rotation in patients undergoing RSA. These postural and biomechanical alterations may have critical implications for surgical planning and postoperative outcomes. Preoperative assessment of sagittal spinal alignment, particularly thoracic kyphosis, should be integrated into the planning process for RSA to optimize implant positioning and improve functional results.

## 1. Introduction

Reverse shoulder arthroplasty (RSA) has become a widely accepted treatment for degenerative shoulder conditions, particularly in elderly patients with rotator cuff arthropathy [1,2,3,4]. While RSA reliably improves active abduction and forward elevation, limitations in internal and external rotation often persist, negatively impacting patients’ ability to perform daily activities [5,6,7,8]. These deficits are not solely related to implant design but are also influenced by the patient’s posture and scapulothoracic orientation [9,10,11]. Scapular internal rotation (SIR), anterior tilt, and protraction—common findings in elderly individuals with thoracic kyphosis—alter the positioning of the glenoid component relative to the humerus, thereby affecting the biomechanics of shoulder movement after RSA [12,13,14]. The posture and motion of the spine directly influence scapular orientation [14]. The increase in thoracic kyphosis, in particular, has been associated with increased scapular internal rotation and anterior scapular tilt. This kyphosis-driven scapular posture effectively reorients the glenoid plane relative to the humerus. Intraoperatively, it can hinder glenoid exposure and make it harder to achieve the intended version, inclination, and lateralization of the components; postoperatively, it alters deltoid and cuff/tendon tensioning, promotes impingement and scapular notching, and limits the arcs of external and internal rotation. Collectively, these effects complicate optimal component alignment and may translate into constrained range of motion (ROM), suboptimal function, and a higher risk of mechanical complications [14,15,16,17]. Recent evidence suggests that posture-related changes in scapular orientation may significantly influence both simulated and clinical outcomes after RSA. In a large retrospective study, Moroder et al. categorized patients into posture types (A, B, and C) based on scapular internal rotation, and demonstrated that those with kyphotic posture (Type C) exhibited significantly reduced abduction, increased pain scores, and worse SPADI and Constant scores at 2-year follow-up [14,18,19]. Furthermore, Siegert et al. confirmed that SIR can be reliably assessed on standard 2D CT scans, even without full visualization of the spine, suggesting its feasibility for preoperative planning [20].

The clinical implications of scapular orientation extend beyond motion range. Moroder et al. also demonstrated that optimal humeral component retrotorsion varies according to scapular internal rotation, with a higher correction angle required in patients with pronounced scapular protraction and kyphosis [15,21]. These findings suggest that a fixed retrotorsion angle may not be universally appropriate, and that individualization of component positioning could reduce impingement and improve rotational balance [15,21,22].

Considering this, assessing thoracic posture and scapulothoracic orientation preoperatively becomes essential. The radiographic Cobb angle, a simple and widely used measure of sagittal spinal curvature, may serve as a surrogate marker for posture-related scapular alignment [14,20]. However, the direct relationship between radiographic kyphosis and SIR has not yet been thoroughly quantified. This study aims to investigate the influence of preoperative thoracic kyphosis, measured by the Cobb angle, on scapular internal rotation (primary outcome) and anterior scapular tilt (secondary outcome) as assessed by CT imaging in patients undergoing RSA. Our hypothesis is that higher degrees of thoracic kyphosis are associated with increased scapular internal rotation, with relevance for glenoid exposure and component positioning potential consequences for glenoid exposure, component positioning, and postoperative outcomes.

## 2. Materials and Methods

From November 2017 to September 2024, retrospectively collected data of 165 patients who underwent RSA by the senior surgeons of the group (E. F., R. P.), obtained from the Campus Shoulder Study Group database, were selected to participate in the present retrospective cohort study. Preoperative data were collected retrospectively. Inclusion criteria were as follows: (a) primary procedure of RSA, (b) age > 18 years at the operation, (c) patients with preoperative CT scan of the operated shoulder, and (d) patients with preoperative chest x-ray in 2 projections which included the cervicothoracic region and the first lumbosacral vertebrae. Approval for this study was provided by the Campus Bio-Medico Ethical Committee (study no. GR7009). Exclusion criteria were as follows: absence and suboptimal quality of preoperative radiographs and CT scan and being a member of the pediatric population. Indications for surgery were eccentric cuff tear arthropathy, concentric osteoarthritis, or nonfunctional, irreparable cuff tears.

### 2.1. Radiological Analysis

**Figure 1:**
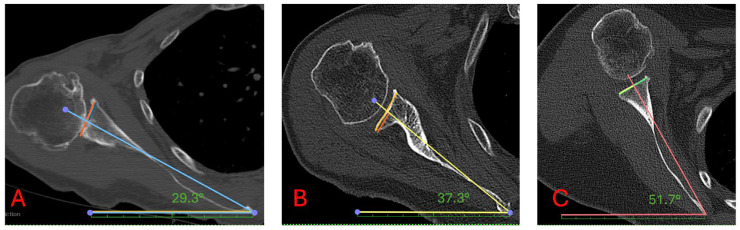
Measurement of the Scapular Internal Rotation (SIR) on axial CT reconstructions. Colored lines in each plane depict the anatomical landmarks and reference axes used to compute the inclination angle [20]. Image (**A**) shows an angle of 29.3°, image (**B**) shows an angle of 37.3°, and image (**C**) shows an angle of 51.7°.

Preoperative CT scans and Chest X-rays of all eligible patients were reviewed by one fellowship-trained shoulder surgeon and two orthopedic residents; then, they were exported, anonymized, and using Horos software 4.0.1 (https://horosproject.org/; Nimble Co LLC d/b/a Purview in Annapolis, MD, USA) or in DICOM (Digital Imaging and Communications in Medicine) format to collect the SIR and Anterior tilt. SIR was defined as an angle between a perpendicular line to the best-fit sagittal axis (midpoint from the vertebra body Th1 and midpoint of the sternum) and a line from the medial root of the scapular spine (trigonum scapulae) to the center of the glenoid (Figure 1) [20]. Anterior scapular tilt was defined as an angle between the midpoint of the glenoid and the inferior angulus and a line drawn vertically along the fixed examination table on a parasagittal view. For independent determination of 2D scapular internal rotation, CT scans were cropped on an axial view, leaving only the scapula and humerus from the acromion to the level of the inferior angulus and excluding visualization of the spine, comparable to a standard clinical CT scan field of view. Internal scapular rotation was then measured at the level of the deepest glenoid concavity as the angle between the midpoint of the glenoid to the medial root of the scapular spine and the standardized CT reference line for the transversal axis. Anterior scapular tilt was evaluated on an en-face glenoid projection; measurements were referenced to the examination table to represent the coronal body axis. Each patient (*n* = 165) was categorized into three different Kyphosis ranges (based on the Cobb angle); the following threshold values were used: A ≤ 36°, B > 36° to 46°, and C ≥ 47°. This grouping was derived from previous internal analyses within our research group, where the Constant score was used as the clinical outcome variable. The cut-off points were identified using maximally selected rank statistics, a statistical method that systematically tests multiple potential cutpoints in a continuous variable to find those that maximize differences in a clinical outcome. This approach aims to establish clinically meaningful thresholds rather than relying solely on arbitrary radiographic criteria. For the Cobb angle, we used a lateral chest radiograph. Anatomical landmarks were identified as follows: the sternoclavicular joint to localize the T2 vertebra, and the aortic arch to identify the vertebral body of T4. The Cobb angle was determined as the angle between the superior endplate of T4 and the inferior endplate of T12, using the anterior and posterior morphometry points of each to define lines parallel to the endplates. Data collection then included age at surgery, height, weight, body mass index (BMI), anterior scapular tilt, SIR, and Cobb angle.

### 2.2. Data Analysis

Descriptive statistics were performed to describe the mean, range, and standard deviation for all variables. All data analyses were performed using STATA 18 Software (StataCorp LLC, College Station, TX, USA). Kruskal–Wallis test was performed to compare Anterior scapular tilt and SIR among groups stratified by Cobb angle. Correlations were performed using the Spearman correlation coefficient. The significance level was set at *p* = 0.05. Post hoc power analysis, as calculated by gPower software 3.1.9.7, indicated that the study sample would allow 82% power in detecting a mean difference of 12 ± 8.1.

## 3. Results

A total of 165 patients who underwent primary reverse shoulder arthroplasty (RSA) were included in the final analysis. The cohort had a mean age of 70.4 years (standard deviation [SD] 9.5), with a predominance of female patients, representing 68.9% of the sample. The right shoulder was the affected side in 70.1% of cases. The mean body mass index (BMI) across the cohort was 28.0 kg/m^2^ (SD 6.3) (Table 1), consistent with a moderately overweight population.

### 3.1. Radiographic Measurements

Radiographic assessment revealed a mean thoracic Cobb angle of 40.7° (SD 12.4), indicating a wide spectrum of sagittal spinal curvature within the cohort. The average SIR was 41.9° (SD 8.4), and the mean anterior scapular tilt measured 24.1° (SD 13.3) (Table 2).

### 3.2. Group Comparisons

**Figure 2:**
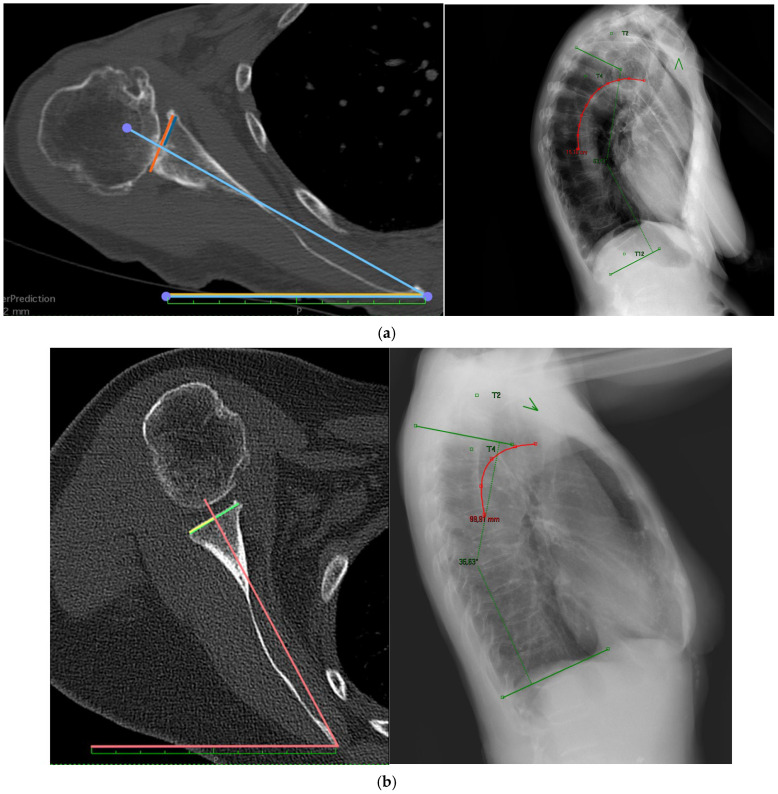
Examples of scapular inclination angle (Scapular Inclination Rotation, SIR) measurement in two patients with varying degrees of scapular tilt. (**a**) The measured SIR is 29.3°, and the corresponding Cobb angle is 53.43° (Type C). (**b**) The SIR is 51.7°, and the Cobb angle is 35.83° (Type A). Axial and sagittal CT reconstructions were used to identify anatomical landmarks and compute angular values.

Analysis across these stratified groups demonstrated a progressive increase in scapular internal rotation with greater degrees of thoracic kyphosis (Figure 2). Specifically, mean SIR was 40.3° (SD 9.3) in Group A, 41.8° (SD 7.8) in Group B, and 43.7° (SD 8.0) in Group C (Table 3). This trend reached statistical significance (*p* = 0.047). Kruskal–Wallis test revealed that the differences between Group A and Group C (*p* = 0.009), as well as between Group B and Group C (*p* = 0.040), were statistically significant. However, the difference between Group A and Group B did not achieve significance (*p* = 0.223), suggesting that the most notable changes in scapular internal rotation are observed in individuals with more severe kyphosis. In contrast, no statistically significant differences were found between groups for anterior scapular tilt (*p* = 0.570). The average tilt angle was 25.8° (SD 13.0) in Group A, 23.3° (SD 12.5) in Group B, and 23.5° (SD 14.8) in Group C. These findings suggest that, unlike SIR, anterior scapular tilt is not strongly influenced by thoracic curvature as measured by the Cobb angle.

### 3.3. Correlation Analyses

Spearman correlation analysis across the entire cohort revealed a weak but statistically significant positive correlation between Cobb angle and SIR (R = 0.24, *p* = 0.002). The coefficient of determination (R^2^ = 0.06) indicated that only a small proportion of the variability in SIR could be explained by the Cobb angle alone. When examining correlations within each kyphosis group, the relationship between Cobb angle and SIR remained weak and failed to reach statistical significance:

- In Group A, the correlation coefficient was R = 0.235 (*p* = 0.094),
- In Group B, R = 0.046 (*p* = 0.718),
- In Group C, R = 0.158 (*p* = 0.278).

No significant correlations were identified between Cobb angle and anterior scapular tilt in any group. Furthermore, no meaningful correlation was found between anterior scapular tilt and SIR, confirming that scapular tilt is relatively independent of sagittal spinal curvature and is likely influenced by other factors.

### 3.4. Demographic Influences

A secondary analysis of demographic parameters revealed a very weak but statistically significant positive correlation between patient age and scapular internal rotation (R = 0.155, *p* = 0.048), suggesting a modest association between increasing age and increased internal scapular orientation. No statistically significant correlations were observed between BMI and any of the radiological parameters assessed, including Cobb angle, SIR, or scapular tilt.

## 4. Discussion

This study provides new insights into the relationship between thoracic kyphosis and scapular internal rotation (SIR) in patients undergoing reverse shoulder arthroplasty (RSA), highlighting the biomechanical implications of postural changes on scapulothoracic orientation and implant planning. By deepening the understanding of posture-related biomechanical alterations, this work supports a patient-specific approach to RSA planning. The integration of spinal alignment into the preoperative assessment may help optimize implant placement, improve functional outcomes, and minimize the risk of complications in reverse shoulder arthroplasty. While the role of thoracic posture in influencing shoulder mechanics has been conceptually recognized, this study offers one of the few quantitative analyses of the association between radiographic kyphosis, as measured by the Cobb angle, and scapular alignment in a surgical population. Our results revealed a statistically significant but weak positive correlation between the Cobb angle and SIR (R = 0.24, *p* = 0.002), consistent with previous observational data suggesting that progressive kyphotic deformity is accompanied by increased scapular internal rotation [16]. When patients were stratified into three kyphosis groups (≤36°, 36–46°, ≥47°), a significant increase in mean SIR was observed across groups, rising from 40.3° in patients with minimal kyphosis to 43.7° in those with severe kyphosis (*p* = 0.047). In contrast, anterior scapular tilt did not exhibit a consistent association with kyphosis in this cohort. These findings are consistent with the postural classification described by Moroder et al., in which patients are categorized into Types A, B, and C according to progressively increasing degrees of scapular internal rotation. Type C patients—marked by scapular protraction and a kyphotic posture—exhibited significantly reduced abduction, greater pain intensity, and poorer functional outcomes (as measured by SPADI and Constant scores) at two-year follow-up [16]. Notably, thoracic kyphosis was not included as a defining criterion in this classification system, suggesting that scapular alignment was evaluated independently of sagittal spinal morphology. Importantly, these scapular adaptations are not merely geometric but have substantial clinical relevance. As confirmed by Moroder et al. (2020), the degree of scapular internal rotation directly influences the optimal retrotorsion angle of the humeral component in RSA [23]. From a practical standpoint, increasing kyphosis may complicate glenoid exposure and component targeting (version/inclination/lateralization) and may contribute to restricted rotational arcs after RSA. These observations support exploring patient-specific planning, including the possibility of individualizing humeral retrotorsion according to measured SIR and emphasizing perioperative rehabilitation focused on scapular mechanics. In patients with more pronounced scapular protraction and thoracic kyphosis, a greater correction angle is required to achieve neutral glenosphere-humerus opposition and preserve functional rotation. This underscores the inadequacy of a “one-size-fits-all” approach and supports a patient-specific strategy for component positioning to reduce impingement and optimize rotational balance. Furthermore, although previous studies have largely relied on full-body 3D CT scans to assess scapulothoracic orientation, our study confirms the feasibility of using standard axial 2D CT scans to estimate SIR, even in the absence of full spinal visualization. This is corroborated by Siegert et al. (2023), who found that SIR measurements obtained from limited-field 2D CT scans were consistent with those from 3D reconstructions, with a mean difference of less than 1° and a maximum deviation of 10.5° [20]. This mixed-modality approach reflects clinical practice but introduces heterogeneity (standing vs. supine, static acquisitions). Such methodological validation is clinically valuable, as it allows routine preoperative imaging to inform component alignment strategies without additional radiation exposure or resource-intensive protocols. Future studies should report intra-/inter-observer reliability (e.g., ICC, Bland–Altman) and consider posture-sensitive, low-dose imaging protocols to harmonize reference frames. Despite these promising findings, it is important to emphasize that the direct biomechanical relationship between Cobb angle and SIR remains poorly defined. While both variables appear to be linked, and the Cobb angle could serve as a surrogate marker for posture-related scapular malalignment, our data show that the correlation is weak (R^2^ = 0.06), indicating that kyphosis accounts for only a limited proportion of SIR variability. This suggests that other factors—including thoracic flexibility, muscular compensation (serratus anterior tone, latissimus dorsi tightness), scapular kinematics during arm elevation, and individual anatomical variability—likely modulate the scapular position beyond static radiographic kyphosis alone. This limitation is echoed in recent scoping reviews, which advocate for multidimensional models incorporating sagittal balance, spine mobility, and patient-specific kinematic profiles [14,24]. In clinical practice, these findings call for a more comprehensive and individualized preoperative assessment. In addition to routine radiographs and CT scans, consideration should be given to posture type (as per Moroder classification), dynamic scapular behavior, and spine alignment. The integration of these variables may improve surgical accuracy and long-term outcomes in RSA. Particularly in elderly patients with age-related postural adaptations, individualized humeral retroversion and careful planning of glenoid component orientation should be prioritized to prevent early impingement, increase range of motion, and improve functional recovery [25,26,27]. These findings further reinforce the clinical rationale for integrating sagittal spinal alignment into the preoperative evaluation of candidates for reverse shoulder arthroplasty (RSA). Recognizing the interplay between thoracic posture and scapular orientation offers an opportunity to enhance surgical planning and patient-specific decision-making. Moving forward, future research should focus on the development of robust and predictive models capable of quantifying the relationship between the Cobb angle and scapular alignment. Additionally, it would be valuable to investigate whether dynamic assessments of posture—such as standing lateral spine radiographs or EOS imaging—offer superior predictive accuracy for postoperative outcomes compared to conventional static imaging in the supine position. Lastly, the clinical relevance of individualized humeral component retrotorsion should be validated through prospective trials, using functional endpoints including shoulder strength, range of motion (ROM), and patient-reported outcome measures. Such efforts could pave the way toward a more precise, posture-informed approach to RSA. Such research would allow for a paradigm shift toward personalized shoulder arthroplasty, in which both osseous anatomy and global posture are factored into surgical planning. This study offers important contributions to understanding the relationship between thoracic kyphosis with scapular internal rotation and anterior tilt; however, several limitations must be acknowledged. First, kyphotic curvature was assessed using preoperative chest radiographs, which may not capture spinal alignment as precisely as computed tomography (CT). While CT imaging could provide more accurate and comprehensive three-dimensional evaluations of thoracic morphology and its biomechanical implications for shoulder function, its use must be balanced against the risks of increased radiation exposure. Second, the representativeness of the underlying cohort is limited, and since the results came from a two-surgeon patient cohort, a larger and more heterogeneous sample would enhance the overall quality and generalizability of the study. Future research should aim to investigate the clinical implications of these findings and assess how clinical outcomes vary in relation to these parameters in patients undergoing reverse shoulder arthroplasty.

## 5. Conclusions

Patients with greater kyphotic curvature exhibited higher SIR values, supporting the influence of sagittal spinal alignment on scapular orientation. However, the low predictive value suggests that the Cobb angle alone cannot reliably predict scapular rotation and must be integrated with other clinical variables. Anterior scapular tilt showed no correlation with kyphosis, indicating it reflects different biomechanical dynamics. This study reinforces the value of posture-informed RSA planning and highlights the need for future research to validate whether incorporating spinal alignment into surgical strategy improves functional outcomes.

## Figures and Tables

**Table 1:** Demographic characteristics.

Female	113 (68.9%)
Age	70.4 (9.5)
BMI	28.0 (6.3)
Right shoulder	115 (70.1%)
BMI: Body Mass Index; mean (SD)	28.0 kg/m^2^ (SD 6.3)

**Table 2:** Radiological characteristics. Mean (SD).

Cobb Angle	Scapular Internal Rotation	Anterior Scapular Tilt
40.7 (12.4)	41.9 (8.4)	24.1 (13.3)

**Table 3:** Radiological comparison between groups.

Cobb Angle	Scapular Internal Rotation (Mean (SD))	*p*-Value	Anterior Scapular Tilt (Mean (SD))	*p*-Value
≤36	40.3 (9.3)	0.047	25.8 (13.0)	0.570
>36–46	41.8 (7.8)		23.3 (12.5)	
≥47	43.7 (8.0)		23.5 (14.8)	

## Data Availability

All relevant data are presented in the article.

## Abbreviations

The following abbreviations are used in this manuscript:

RSA	Reverse shoulder arthroplasty
SIR	Scapular internal rotation
SD	Standard deviation
BMI	Body mass index
